# Mimosine Dipeptide Enantiomsers: Improved Inhibitors against Melanogenesis and Cyclooxygenase

**DOI:** 10.3390/molecules200814334

**Published:** 2015-08-06

**Authors:** Binh Cao Quan Nguyen, Shinkichi Tawata

**Affiliations:** 1 Department of Bioscience and Biotechnology, The United Graduate School of Agricultural Sciences, Kagoshima University, Korimoto 1-21-24, Kagoshima 890-8580, Japan; E-Mail: ncqbinh@gmail.com; 2 Department of Bioscience and Biotechnology, Faculty of Agriculture, University of the Ryukyus, Senbaru 1, Nishihara-cho, Okinawa 903-0213, Japan

**Keywords:** mimosine, mimosine dipeptide, melanogenesis, cyclooxygenase

## Abstract

Melanogenesis plays an important role in the protection of skin against UV through production of melanin pigments, but abnormal accumulation of this pigment causes unaesthetic hyperpigmentation. Much effort is being made to develop effective depigmenting agents. Here, we show for the first time that a small library of mimosine dipeptide enantiomers (Mi-l/d-amino acid) inhibit the melanogenesis in B16F10 melanoma cells by down-regulating the cellular tyrosinase with little effect on their growth or viability. Two of them, Mi-d-Trp and Mi-d-Val, turned out to be the most potent inhibitors on melanin content and cellular tyrosinase in B16F10 melanoma cells. In addition, most of the mimosine dipeptides were more potent than mimosine for inhibiting cyclooxygenase 1 (COX-1) with IC_50_ of 18–26 μM. Among them, Mi-l-Val and Mi-l-Trp inhibited cyclooxygenase 2 (COX-2) more potently than indomethacin, with IC_50_ values of 22 and 19 μM, respectively. Taken together, our results suggest the possibility that mimosine dipeptides could be better candidates (than mimosine) for anti-melanogenic (skin hyperpigmentation treatment) and cyclooxygenase (COX) inhibition.

## 1. Introduction

Melanogenesis is a physiological process that results in the synthesis of melanin pigment and has many functions in living systems [1]. Melanin is an important factor for protection of skin against deleterious UV irradiation. However, the excessive production of melanin causes hyperpigmentation [2] that is a skin pigmentation disorder in which skin becomes darker in color compared to the normal surrounding skin [3]. Tyrosinase is a key enzyme in melanogenesis in melanocytes [4]. It acts as the catalyst for two rate-limiting steps of melanogenesis, the hydroxylation of tyrosine to 3,4-dihydroxyphenylalanine (DOPA) and oxidation of DOPA to dopaquinone [5]. Therefore, the inhibition of tyrosinase activity is capitalized for inhibition of melanogenesis and the treatment of skin hyperpigmentation and whitening [2].

Nonsteroidal anti-inflammatory drugs (NSAIDs) have many therapeutic benefits in the treatment of rheumatoid arthritis and other inflammatory conditions [6]. The major action of NSAIDs is based on inhibition of cyclooxygenase (COX), which is the rate limiting enzyme in the pathway facilitating the conversion of arachidonic acid to inflammatory prostaglandins [7]. There are two different isoforms of COX, COX-1 and COX-2 [8]. COX-1 is expressed in essentially all mammalian tissues, and is responsible for prostaglandin production that maintains homeostasis. In contrast, COX-2 is manifested constitutively in brain, kidneys, and ovaries, and is activated in cells in response to inflammatory stimuli [9,10].

Mimosine [*β*-[*N*-(3-hydroxy-4-oxypyridyl)]-*α*-aminopropionic acid] is a non-protein amino acid containing an alanine side chain bound to the nitrogen atom of a pyridine ring. It is found in several tropical and subtropical plants, which possesses a wide range of biological activities [11,12]. It is hypothesized that mimosine could be used as a source of bioactive compounds and could be useful in the design of novel drugs. Recently, we reported on a series of mimosine tetrapeptides, which are more effective than mimosine to inhibit neuraminidase and tyrosinase as well as the oncogenic/ageing kinase PAK1 [12,13]. Interestingly, melanogenesis and COX activation are among a variety of PAK1-dependent phenomena [13]. Thus, in continuation of our earlier work, we synthesized a small library of enantiomeric mimosine dipeptides and found that they inhibit at least COX and melanogenesis in B16F10 melanoma cells more effectively than mimosine.

## 2. Results and Discussion

### 2.1. Synthesis and Tyrosinase Inhibition of Mimosine Dipeptides

**Figure 1:**
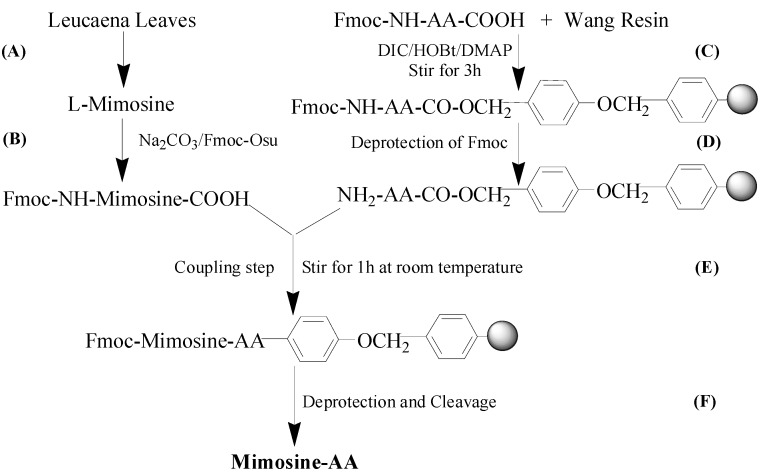
General route of mimosine dipeptide synthesis. (**AA**) l- or d-amino acid; (**A**) l-mimosine isolation from *Leucaena leucocephala* leaves using ion-exchange resin; (**B**) Preparation of Fmoc-mimosine; (**C**) Attachment of Wang resin to Fmoc-amino acid; (**D**) Deprotection of Fmoc using 25% piperidine; (**E**) Coupling of Fmoc-mimosine and amino acid-resin mixture along and the Kaiser test; (**F**) Deprotection and cleavage using 95% trifluoroacetic acid (TFA) to afford desired mimosine dipeptides.

**Figure 2:**
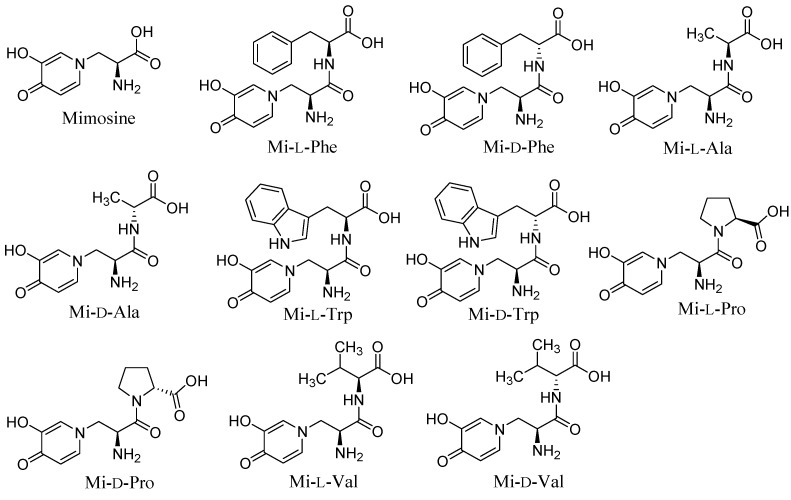
The chemical structures of mimosine and mimosine dipeptides.

It has been reported that the attachment of amino acids to kojic acid has generated compounds that strongly inhibit tyrosinase [14]. Like kojic acid, mimosine is a tyrosine analog and could potentially be used for designing more potent tyrosine inhibitors [12]. Thus, we developed mimosine dipeptides and assessed their ability to inhibit tyrosinase. Our general strategy for the design of these compounds was based on the conjugation of mimosine and an amino acid through solid-phase synthesis using Fmoc chemistry. We synthesized ten mimosine dipeptides using enantiomeric amino acids, including phenylalanine (Phe), alanine (Ala), valine (Val), tryptophan (Trp), and proline (Pro). As shown in Figure 1, l- or d-amino acids were attached to Wang resin support. Mimosine was converted to Fmoc-mimosine which was then coupled to the amino acid to obtain the desired mimosine dipeptides. The chemical structures of mimosine and mimosine dipeptides are shown in Figure 2.

The *in vitro* assay for tyrosinase inhibition was performed using l-tyrosine as a substrate. As expected, the synthesized mimosine dipeptides inhibited tyrosinase more potently than mimosine (Table 1). In particular, conjugation of tryptophan, valine, and proline or of a d-form amino acid to mimosine led to stronger tyrosinase inhibition. Of the four most potent inhibitors, the IC_50_ values of Mi-l-Pro and Mi-d-Trp were 13 and 17 μM, respectively. The IC_50_ of Mi-l-Val and Mi-d-Val against tyrosinase was 12 and 10 μM, marginally lower than that of the positive control, kojic acid (14 μM).

**Table 1:** IC_50_ values of mimosine and their dipeptides for mushroom tyrosinase inhibition.

Compound	IC_50_ (μM) Mushroom Tyrosinase Inhibition
Mi-l-Phe	24.1 ± 3.8 ^ab^
Mi-d-Phe	19.7 ± 0.8 ^bc^
Mi-l-Ala	23.1 ± 2.2 ^b^
Mi-d-Ala	17.7 ± 3.1 ^bc^
Mi-l-Pro	13.1 ± 3.3 ^bc^
Mi-d-Pro	17.2 ± 2.1 ^bc^
Mi-l-Val	11.5 ± 0.5 ^c^
Mi-d-Val	10.3 ± 0.4 ^c^
Mi-l-Trp	19.2 ± 1.5 ^bc^
Mi-d-Trp	16.9 ± 2.1 ^bc^
Mimosine	32.4 ± 1.1 ª
Kojic acid	13.7 ± 1.5 ^bc^

Different letters in the same column indicate the existence of significant difference statistically. Values represented as mean ± SE.

**Figure 3:**
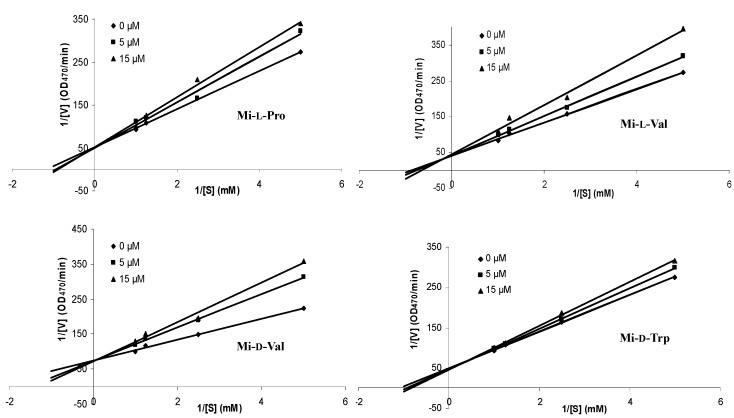
Effect of Mi-l-Pro, Mi-l-Val, Mi-d-Val, and Mi-d-Trp on tyrosinase activity. Tyrosinase activity was assayed as described in the text. Lineweaver-Burk plots were drawn in the presence of the compounds at various concentrations (0, 5, and 15 μM) with different concentrations of the substrate l-tyrosine.

Because of their potent inhibition of tyrosinase, we carried out a kinetic analysis of these four compounds. Lineweaver-Burk plots of 1/[V] versus 1/[S] are shown in Figure 3. The kinetic study of tyrosinase inhibition by these selected compounds revealed that the K_m_ value was increased as the concentration of inhibitors was increased, while the V_max_ values remained unchanged. Therefore, like mimosine, these compounds are considered to be competitive inhibitors of tyrosinase [15]. The K_i_ value of these compounds are shown in Table 2. The inhibition constant of Mi-d-Val (0.02 mM) was less than that of Mi-l-Val, Mi-l-Pro, Mi-d-Trp, suggesting that Mi-d-Val has the most potent inhibitory effect.

**Table 2:** Kinetics and inhibition constant of four selected mimosine dipeptides on the activity of mushroom tyrosinase.

Compound	Inhibition Type	Inhibition Constant (K_i_) (mM)
Mi-l-Pro	competitive	0.05
Mi-l-Val	competitive	0.03
Mi-d-Val	competitive	0.02
Mi-d-Trp	competitive	0.08

### 2.2. Inhibition of Melanogenesis by Mimosine Dipeptides

**Figure 4:**
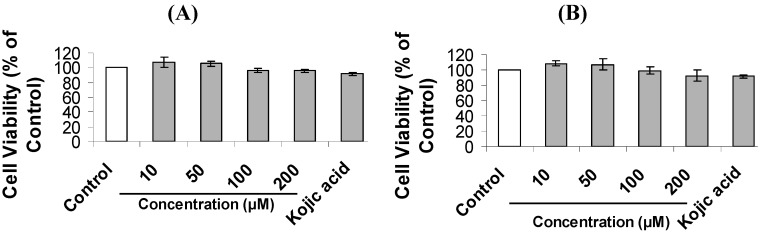

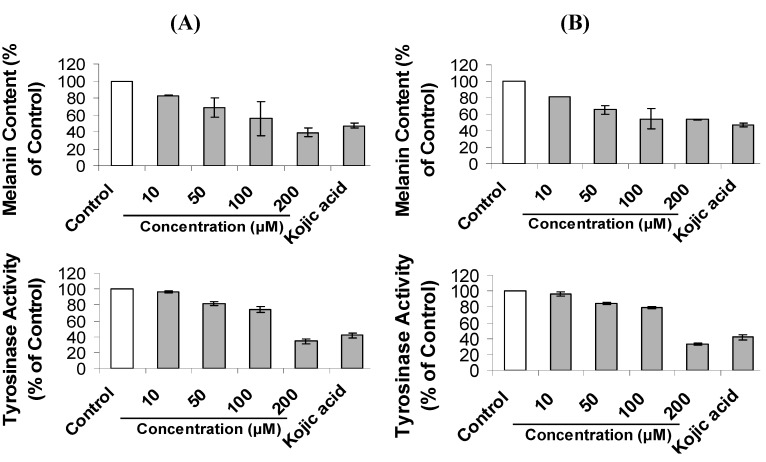
Reduction of the most potent dipeptides on melanin content and cellular tyrosinase in B16F10 cell. (**A**) Mi-d-Trp; (**B**) Mi-d-Val. Cells were treated with various concentrations of tested compounds for 48 h. Results are expressed as percentage in comparison with control. Kojic acid (500 μM) was used as positive control. The experiments were conducted with three replications per each treatment with two independent experiments, and the values are means ± SE. Data have statistical significance at *p* ≤ 0.01.

As mentioned above, we showed that a small library of mimosine dipeptides had strong tyrosinase inhibition *in vitro*. Thus, we hypothesized that these compounds could exhibit melanogenesis inhibition in B16F10 melanoma cells. First, we examined the effect of mimosine dipeptides on cell survival by the thiazolyl blue tetrazolium bromide (MTT) assay, and found that none of them exhibited any significant cytotoxicity up to 100 μM. To determine the anti-melanogenic activity, we measured their effect on the melanin content in B16F10 cells. Generally, mimosine dipeptides reduced the melanin content more effectively than mimosine. Four of them (Mi-l-Pro, Mi-d-Val, Mi-d-Trp, Mi-l-Trp) exhibited strong inhibition on melanin content, with IC_50_ values ranging 109-116 μM, compared with mimosine (IC_50_ around 226 μM) (Table 3). To examine the possible mechanism underlying their anti-melanogenic act, cellular tyrosinase activity was evaluated. The results indicated that all of them strongly inhibited intracellular tyrosinase activity with IC_50_ values ranging from 145–194 μM when compared to mimosine (IC_50_ around 388 μM) (Table 3). Mi-d-Trp and Mi-d-Val are the most potent inhibitors on melanin content and cellular tyrosinase in B16F10 melanoma cells (Figure 4). Taken together, their anti-melanogenic effect exerted by their cellular tyrosinase suppression in B16F10 cells. As shown in Table 3, all prepared mimosine dipeptides had more anti-melanogenic activity than mimosine. When the C-terminus of mimosine was converted to the amide form, anti-melanogenic activity became better than that of the free acid form, suggesting the importance of *C*-terminal amide form in mimosine-peptide conjugates.

**Table 3:** IC_50_ of mimosine dipeptides against intracellular tyrosinase and melanin content in B16F10 melanoma cells.

Compound	IC_50_ (μM) for Cellular Tyrosinase Inhibition	IC_50_ (μM) for Melanin Content Inhibition
Mi-l-Phe	179.2 ± 1.3 ^cd^	120.6 ± 8.6 ^de^
Mi-d-Phe	194.5 ± 6.2 ^b^	168.4 ± 1.4 ^c^
Mi-l-Ala	172.2 ± 4.6 ^cd^	128.0 ± 7.4 ^d^
Mi-d-Ala	180.9 ± 6.8 ^bc^	199.0 ± 5.2 ^b^
Mi-l-Pro	166.8 ± 7.3 ^def^	110.4 ± 5.3 ^e^
Mi-d-Pro	145.9 ± 5.2 ^h^	123.3 ± 3.0 ^de^
Mi-l-Val	172.1 ± 6.5 ^de^	170.5 ± 7.5 ^c^
Mi-d-Val	162.1 ± 4.1 ^efg^	112.2 ± 8.1 ^e^
Mi-l-Trp	156.4 ± 8.3 ^fg^	109.3 ± 2.7 ^e^
Mi-d-Trp	150.0 ± 2.3 ^gh^	116.3 ± 4.9 ^de^
Mimosine	388.4 ± 7.2 ^a^	225.7 ± 4.0 ^a^

Different letters in the same column indicate the existence of significant difference statistically. Values represented as mean ± SE.

The RAC/CDC42-activated kinase 1 (PAK1) is responsible for a variety of diseases/disorders including cancers, neurofibromatosis (NF), diabetes (type 2), hypertension, short lifespan and neurodegenerative diseases such as Alzheimer’s (AD) and Huntington’s disease. Since PAK1 is not essential for the growth of normal cells, blocking PAK1, *per se*, does not cause any side effect [16]. Recently, we demonstrated that PAK1 is essential for the melanogenesis in skin cells such as the melanoma B16F10 [17]. Moreover, in our previous study, three mimosine tetrapeptides, which directly inhibit oncogenic/kinase PAK1 [13], are anti-melanogenic [18]. Thus, it is most likely that mimosine dipeptides also could inhibit melanogenesis by blocking PAK1. We are planning to test whether mimosine dipeptides inhibit PAK1 in B16F10 cells or not.

### 2.3. Inhibition of Cyclooxygenase (COX) by Mimosine and Their Dipeptides

During screening for novel targets of mimosine, we found that cyclooxygenase (COX) was inhibited by mimosine. As shown in Table 4, mimosine inhibited COX-1 and COX-2 with IC_50_ values of 29 and 21 μM, respectively. These results indicated that mimosine inhibits COX-2 more potently than COX-1. Mimosine was also a more potent inhibitor of COX-2 than indomethacin (IC_50_ = 28 μM). Zebardast *et al*. have reported that inhibition of gastroprotective prostaglandins synthesis through COX-1 pathway will lead to the gastrointestinal side effects associated with NSAIDs [19]. Therefore, selective inhibition of COX-2 over COX-1 may be useful for treating inflammation and related disorders, as well as reducing gastrointestinal toxicities. Moreover, mimosine inhibits the transcription and translation of MIP-2 and MCP-1, which can induce inflammatory diseases through the recruitment of neutrophils and mononuclear cells, respectively [20]. These data, in addition to our results, suggest that mimosine may be a promising anti-inflammatory compound.

Because mimosine inhibits cyclooxygenases, the effect of mimosine dipeptides on these enzymes was also explored. We found that most of the synthesized dipeptides were more potent inhibitors of COX-1 than mimosine. The IC_50_ values of the six investigated compounds ranged 18–26 μM as compared with mimosine (29 μM). Mi-l-Val and Mi-l-Trp inhibited COX-2 more potently than indomethacin, with IC_50_ values of 22 and 19 μM, respectively. In both the COX-1 and COX-2 assay, Mi-l-Trp was the most potent inhibitor among the synthesized dipeptides.

**Table 4:** IC_50_ values of mimosine and their dipeptides for cyclooxygenase (COX) isoenzymes inhibition.

Compound	Yield (mg)	IC_50_ (μM) *^ii^*		SI *^i^* COX-1/COX-2
		COX-1	COX-2	
Mi-l-Phe	132.0	nt	nt	nt
Mi-d-Phe	48.5	nt	nt	nt
Mi-l-Ala	45.0	nt	nt	nt
Mi-d-Ala	79.0	19.1 ± 1.0 ^b^	198.4 ± 3.7 ^a^	0.10
Mi-l-Pro	105.5	17.9 ± 2.1 ^b^	29.3 ± 3.0 ^c^	0.75
Mi-d-Pro	43.0	21.0 ± 2.0 ^ab^	127.5 ± 7.7 ^b^	0.15
Mi-l-Val	17.0	24.4 ± 1.4 ^ab^	21.7 ± 2.0 ^c^	1.12
Mi-d-Val	25.0	nt	nt	nt
Mi-l-Trp	98.0	20.4 ± 1.6 ^ab^	19.3 ± 1.8 ^c^	0.95
Mi-d-Trp	71.5	26.1 ± 1.3 ^ab^	27.5 ± 2.2 ^c^	0.95
Mimosine		28.8 ± 2.3 ^a^	20.9 ± 2.1 ^c^	1.38
Indomethacin		6.1 ± 0.9 ^c^	27.7 ± 1.9 ^c^	0.22

*^i^* SI: selectivity index (COX-1 IC_50_/COX-2 IC_50_). *^ii^* Different letters in the same column indicate the existence of significant difference statistically. nt: not tested. Values represented as mean ± SE.

## 3. Experimental Section

### 3.1. Chemicals and Reagents

Fmoc-l-amino acids were purchased from Hipep Laboratories (Kyoto, Japan) whereas Fmoc-d-amino acids were obtained from Sigma-Aldrich (Tokyo, Japan). *N*-[9-fluorenylmethoxycarbonyloxy]succinimide (Fmoc-Osu), 2-[1*H*-benzotriazole-1-yl]-1,1,3,3-tetramethyluronium hexafluorophosphate (HBTU) were from Novabiochem (Hohenbrunn, Germany). Wang resin (1% DVB), *N*,*N*ʹ-diisopropylcarbodiimide (DIC), *N*,*N*ʹ-diisopropylethylamine (DIPEA), and 1-hydroxy-1*H*-benzotriazole (HOBt), Dulbecco’s modified minimum essential medium (D-MEM) were purchased from Wako Pure Chemical Industries (Osaka, Japan). Thiazolyl blue tetrazolium bromide (MTT) were obtained from Sigma-Aldrich (Tokyo, Japan). Fetal bovine serum (FBS) was purchased from HyClone Laboratories Inc. (Victoria, Australia). Penicillin/streptomycin was obtained from Lonza Walkersville, Inc. (Walkersville, MD, USA). Mushroom tyrosinase was obtained from Sigma-Aldrich (St. Louis, MO, USA). Unless otherwise mentioned, all reagents used were of analytical grade and were obtained from Wako Pure Chemical Industries, Ltd. and Kanto Chemical Co., Inc., Japan. The ^1^H- and ^13^C-NMR spectra were recorded on a JEOL JNM-ECA400 (JEOL, Tokyo, Japan). Chemical shifts are expressed in parts per million (*δ*) relative to tetramethylsilane (TMS).

### 3.2. Mimosine Isolation from Leucaena leucocephala Leaves

Samples of *Leucaena leucocephala* leaves were collected near the Faculty of Agriculture, University of the Ryukyus, located at 26° N, 127° E. Fresh leaves (1.5 kg) were boiled in 5 L of water for 10 min. The cooled liquid extract was sieved by suction filtration (Shaking Baths SB-20, As One, Osaka, Japan), and the filtrate was mixed with ion-exchange resin (2 kg). After stirring for 30 min, the mixture was incubated overnight. The resin was rinsed with distilled water 5–6 times and added dropwise to 5 L of 80% ethanol to remove the chlorophyll. Mimosine was removed from the resin by dropwise addition of 6 L of 2 N NH_4_OH. The liquid extract was concentrated to a final volume of 300 mL at 40 °C under reduced pressure. The solution was adjusted to pH 4.5–5.0 with 6 N HCl, and mimosine was precipitated at 4 °C overnight. The resulting mimosine was recrystallized using 5 N NaOH (pH 9.0) and 6 N HCl (pH 4.5–5.0), then allowed to stand at 4 °C to form pure mimosine. Mimosine was stored at −20 °C for further analysis [21]. Mimosine was identified by LC-MS (ESI-): *m*/*z* [M + H]^+^ 199.1.

### 3.3. Preparation of Fmoc-Mimosine 

Mimosine (2.5 g) and sodium carbonate (Na_2_CO_3_) (2.75 g) were dissolved in distilled water (37.5 mL). Fmoc-Osu (6.25 g) dissolved in 37.5 mL of 1,4-dioxane was added dropwise to the solution and stirred for 20 h at room temperature. Next, 150 mL of Na_2_CO_3_ (0.1 M) was added. The mixture was stirred for 7 h at 26 °C and was then filtered and washed with 75 mL of ethyl acetate to remove excess Fmoc-Osu and by-products. The water fraction was kept in an ice bath and adjusted to pH 4.0 using 6 N HCl and incubated overnight at 4 °C. The resulting precipitate was filtered, washed with distilled water, and dried under reduced pressure to give Fmoc-mimosine [12].

### 3.4. General Procedure for Synthesis of Mimosine Dipeptides

Fmoc-amino acids (l- or d-form) (2.35 mmol) were dissolved in a solution of HOBt (2.35 mmol, 360 mg) and DIC (2.35 mmol, 365 μL), followed by preactivation in 5 mL of dimethylformamide (DMF). The solution was then added to Wang resin (0.5 g) which was swollen in 2 mL of dichloromethane (CH_2_Cl_2_) for 30 min. 4-Dimethylaminopyridine (DMAP) (0.235 mmol, 29 mg) was then suspended in 500 μL of DMF and added to the mixture prior to stirring for 3 h at room temperature. This procedure was repeated twice, and the resin was washed with DMF, CH_2_Cl_2_, methanol (MeOH), and dried under reduced pressure. A small portion of the resin (5 mg) was used for analysis of Fmoc-group content. Fmoc protection was removed by adding 25% piperidine and shaking for 45 min at room temperature. To couple the mimosine to the amino acids, a solution of Fmoc-mimosine (4 equiv relative to amino acid on resin), HOBt (4 equiv), HBTU (3.6 equiv), and DIPEA (8 equiv) was added to the amino acid-resin suspended in DMF, and the mixture was stirred at room temperature for 1 h. The Kaiser’s test was used to assess the completeness of the coupling reaction. The final cleavage was performed by shaking the resin vigorously in 95% trifluoroacetic acid (TFA) for 90 min and then filtering and washing with TFA. Ice-cold diethyl ether was used to precipitate the combined washings. The resulting precipitate was filtered, washed with diethyl ether, and dried under a vacuum to obtain the desired mimosine dipeptides. All crude peptides were a white solid, and their yields are presented in Table 1. All samples were purified using high-performance liquid chromatography (HPLC) (Shimadzu, Kyoto, Japan) before being used in enzyme inhibition assays. The major peaks were collected using a Phenomenex (150 mm × 4.6 mm; 4 μm) column with mobile phase of solvent A (0.1% TFA in water) and solvent B (0.1% TFA in acetonitrile). The flow rate and absorbance wavelength were set at 1 mL/min and 210 nm, respectively. The purified compounds were identified using LC-MS (ESI): *m*/*z* [M − H]^+^ 344.2, 344.3, 268.1, 268.3, 383.2, 382.3, 296.2, 296.4, 294.1, 294.4 for Mi-l-Phe, Mi-d-Phe, Mi-l-Ala, Mi-d-Ala, Mi-l-Trp, Mi-d-Trp, Mi-l-Val, Mi-d-Val, Mi-l-Pro, and Mi-d-Pro, respectively. Representative data for some of the synthesized mimosine dipeptides are as follows.

Data for Mi-l-Phe. ^1^H-NMR (400 MHz, D_2_O) *δ*: 3.27–3.14 (dd, 2H, CH_2_), 3.80 (s, 1H, CH-NH_2_), 3.99 (q, 1H, CH-NH_2_), 4.17 (q, 1H, CH), 6.59 (d, 1H, CH), 7.33 (s, 1H, CH), 7.38 (s, 1H, CH), 7.43 (s, 1H, CH), 7.65 (s, 1H, CH). ^13^C-NMR *δ*: 36.30, 55.99, 71.12, 97.49, 114.79, 127.65, 129.07, 129.32, 135.05, 140.2, 148.2, 173.89, 181.52. LC-MS (ESI): m/z 344.2 [M − H]^+^.

Data for Mi-l-Ala. ^1^H-NMR (400 MHz, D_2_O) *δ*: 1.48 (d, 3H, CH_3_), 3.78 (dd, 1H, CH-NH_2_), 4.20–4.17 (q, 1H, CH-NH_2_), 4.49–4.45 (q, 1H, CH_2_), 4.50–4.64 (q, 1H, CH), 6.60 (d, 1H, CH), 7.67 (s, 1H, CH). ^13^C-NMR *δ*: 16.08, 42.80, 50.47, 97.79, 114.82, 127.65, 129.07, 130.70, 138.52, 140.2, 148.2, 175.75, 181.55. LC-MS (ESI): m/z 268.1 [M − H]^+^.

Data for Mi-l-Trp. ^1^H-NMR (400 MHz, D_2_O) *δ*: 3.27 (d, 2H, CH_2_), 3.32 (d, 2H, CH_2_), 3.50 (q, 1H, CH-NH_2_), 4.05 (q, 1H, CH-NH_2_), 4.15 (q, 1H, CH), 4.45 (q, 1H, CH), 6.57 (d, 1H, CH), 7.17 (s, 1H, CH), 7.26 (d, 1H, CH), 7.74–7.30 (m, 4H, benzene). ^13^C-NMR *δ*: 26.32, 48.07, 55.00, 68.44, 90.77, 98.99, 107.04, 111.89, 118.39, 119.41, 122.08, 124.97, 132.77, 136.34, 138.31, 148.2, 174.43, 181.52. LC-MS (ESI): m/z 383.2 [M − H]^+^.

### 3.5. Tyrosinase Inhibition Assay

A microplate assay was used to assess tyrosinase inhibition, as previously described [22]. Samples (20 μL) with various concentrations were transferred into each well of 96-well plate, and 120 μL of 20 mM sodium phosphate buffer (pH 6.8), 20 μL of 500 U/mL mushroom tyrosinase enzyme dissolved in buffer were added. The mixture was incubated at 25 °C for 15 min, followed by addition of the reaction substrate 20 μL of 0.85 mM l-tyrosine solution. The absorbance was recorded at 470 nm using a microplate reader (Benchmark plus, Biorad, Herfordshire, UK). Kojic acid was used as the positive control. For the kinetic studies of selected inhibitors, we used a range of l-tyrosine concentrations for each inhibitor concentration. Preincubation and measurement times were the same as above. Lineweaver-Burk plots were used to determine the type of enzyme inhibition, and the inhibition constant K_i_ was determined by plotting K_m_/V_max_ versus the inhibitor concentration. Percent inhibition was calculated using the following equation:

Inhibition (%) = ([C_E_ − C_o_] − [S_E_ − S_o_])/(C_E_ − C_o_) × 100 (1)

where C_E_ is the absorbance of the control samples with enzyme, C_o_ is the absorbance of the control samples without enzyme, S_E_ is the absorbance of the tested sample with enzyme, and S_o_ is the absorbance of the tested sample without enzyme.

### 3.6. Cell Culture

Mouse B16F10 cell line was obtained from American Type Culture Collection (Manassas, VA, USA). Cells were cultured in Dulbecco’s modified minimum essential medium (D-MEM) medium containing 10% fetal bovine serum (FBS), 1% penicillin/streptomycin in a humidified atmosphere with 5% CO_2_ incubator at 37 °C.

### 3.7. Cell Viability Assay

The assay as described by Campos *et al*. [23] is based upon the cleavage of the yellow tetrazolium salt MTT to give purple formazan crystals. Cells were seeded in a 96-well plate at a density 5 × 10^3^ cells/well and cultured for 24 h before the compounds (10–500 μM) were added to the medium containing D-MEM supplemented with 10% FBS, 1% penicillin/streptomycin. Cells were incubated in a humidified atmosphere 5% CO_2_ at 37 °C for 48 h. Afterward, 20 μL of MTT solution (0.5 mg/mL) were added to each well and plates were incubated for 3 h. The medium was removed and formazan was dissolved in 200 μL of DMSO. The plates were shaken for 10 min and cell viability was assessed by measuring the absorbance at 570 nm using microplate reader (Benchmark plus, Biorad, Hertfordshire, UK). Kojic acid (500 μM) was used as positive control. DMSO was used as the blank. Absorbance of sample and control subtracts the absorbance of blank. The corrected absorbance of sample divides the corrected absorbance of control, then multiplies 100% to give the percentage of cell viability.

### 3.8. Determination of Melanin Content in B16F10 Cells

The assay was performed following previously described procedure. Cells (5 × 10^3^ cells/well) were seeded in a 96-well plate and cultured for 24 h. Compounds (10–500 μM) were added and incubated at 37 °C for 48 h. The medium was replaced by a fresh medium containing the same concentration of compound and the cells were incubated for 48 h. The medium was removed and the cells were dissolved in 100 μL of 1 N NaOH. The reaction was heated at 90 °C for 1 h. Melanin content was estimated by the absorbance at 400 nm [24].

### 3.9. Intracellular Tyrosinase Inhibition Assay

Cells were seeded in a 96-well plate at a density 5 × 10^3^ cells/well and the compounds (10–500 μM) were added after 24 h treatment. Cells were incubated for 48 h, washed two times with 50 mM of ice-cold phosphate buffer (pH 6.8), lysed with 90 μL of 50 mM phosphate buffer (pH 6.8) containing 1% Triton-X, and frozen at −80 °C for 30 min. After thawing and mixing, 20 μL of 0.5% l-DOPA was added to each well. The mixture was incubated for 2 h at 37 °C and absorbance was measured at 490 nm [23].

### 3.10. Cyclooxygenase (COX) Inhibition Assay

Inhibition of COX activity was measured using a colorimetric COX (ovine) inhibitor screening assay kit (Cayman Chemical, Ann Arbor, Michigan, USA). First, 150 μL of assay buffer (0.1 M Tris-HCl, pH 8.0), 10 μL of heme, and 10 μL of enzyme (COX-1 or COX-2) were added to an anti-rabbit IgG coated 96-well microtiter plate. Immediately, 10 μL of sample was added to inhibitor wells while 10 μL of solvent or buffer was added to control and background wells, respectively. After incubation for 5 min at 25 °C, 20 μL of the colorimetric substrate solution and 20 μL of 22 mM arachidonic acid were added to all the wells. The mixture was shaken for a few seconds and incubated for an additional 5 min at 25 °C. The absorbance was determined at 590 nm using a microplate reader (Benchmark plus, Biorad, Herfordshire, UK). Indomethacin was used as the positive control. The corrected absorbance of all the samples and controls was calculated by subtracting the absorbance of the background wells. The percentage of inhibition was calculated as follows:

Inhibition (%) = (A_o_ − A_s_)/A_o_ × 100 (2)

where A_o_ and A_s_ are the absorbance of the corrected control and sample, respectively.

### 3.11. Data Analysis

All statistical analyses were performed using statistical analysis system (SAS) software, version 9.1.3 (SAS Institute Inc., Cary, CA, USA). Data were analyzed by one-way analysis of variance (ANOVA) with Duncan’s *post hoc* test (*p* ≤ 0.01). All calculations were performed using Excel, Microsoft Office 2003. The IC_50_ values were determined graphically.

## 4. Conclusions

We provide the first evidence of melanogenesis inhibition of a small library of mimosine dipeptide enantiomers. Their anti-melanogenic activity is due to intracellular tyrosinase inhibition with little effect on the growth or viability of B16F10 melanoma cells. In addition, some of these dipeptides inhibited more strongly COX-1/2 than mimosine. Our results suggest that these new compounds may be the better candidates for anti-melanogenics (skin hyperpigmentation therapeutics) and COX inhibitors.
